# AI enhancing prefabricated aesthetics and low carbon coupled with 3D printing in chain hotel buildings from multidimensional neural networks

**DOI:** 10.1038/s41598-025-97858-8

**Published:** 2025-04-17

**Authors:** Gangwei Cai, Yin Lou, Feidong Lu

**Affiliations:** 1 College of Architecture and Urban Planning, Tongji University, Shanghai, China; 2 Hangzhou International Urbanology Research Center & Zhejiang Urban Governance Studies Center, Hangzhou, China; 3 Baoye Daiwa Industrialized House Manufacturing Co., Ltd., Daiwa House Group, Osaka, Japan; 4 Shaoxing Keqiao District Science and Technology Innovation Service Center, Shaoxing, China; 5 Tongji Architectural Design (Group) Co., Ltd, Shanghai, China

**Keywords:** Artificial intelligence (AI), Low-carbon aesthetic design, Machine learning (ML), Neural networks (NN), Statistical modeling (SM), Prefabricated hotels, Robotic 3D printing, Sustainable tourism, Sustainable development goals (SDGs), Sustainability, Energy management, Energy policy, Energy and society, Energy and behaviour, Civil engineering

## Abstract

There are approximately 70,000 economy chain hotels worldwide, generating about 300 million tons of carbon dioxide annually. While reducing carbon emissions can lower energy consumption, these hotels must also continually attract guests to ensure revenue growth and achieve sustainable development. This study focuses on the application of Artificial Intelligence (AI) in the prefabricated renovation of hotels, investigating how AI plays a crucial role in coupling low-carbon construction and aesthetic design. Using multidimensional algorithms within machine learning (ML), neural networks (NN), and statistical modeling (SM), this paper analyzes the impact of AI-driven prefabricated room renovations on tourist satisfaction and carbon emissions. The results indicate that AI can not only optimize energy consumption and structural efficiency in the renovation process but also achieve low-carbon goals while maintaining high-quality aesthetic designs. This study offers new theoretical insights into the integration of low-carbon and aesthetic design, filling gaps in the current literature, providing a pathway for achieving sustainable development goals (SDG 7, 8, and 12), and offering valuable implications for robotic intelligent construction and 3D printing in prefabricated buildings industry.

## Introduction

In recent years, the hospitality industry has increasingly focused on adopting low-carbon building design and sustainable technologies^1,2^. As part of this trend, many hotels have begun to implement eco-friendly and energy-efficient renovations^3^. However, while the environmental benefits of these initiatives are widely recognized, there is a growing concern that aesthetic considerations may be overlooked in the pursuit of sustainability^4,5^. This issue is particularly pressing in the hotel industry, where guest satisfaction is often influenced by the aesthetic quality of the environment^6^. Therefore, it is crucial to explore innovative solutions that balance both low-carbon goals and aesthetic design in hotel renovations^7^. In response to this challenge, prefabricated hotels—those built using modular, factory-produced components—have gained attention for their potential to integrate both sustainable and aesthetic design features efficiently^8,9^. These hotels are seen as a promising avenue for achieving energy efficiency and sustainability in building construction and renovation while maintaining high-quality design standards^10^. The use of Artificial Intelligence (AI) in the renovation process, specifically in prefabricated hotels, presents an exciting opportunity to further enhance both the aesthetic appeal and the low-carbon performance of these structures^11,12^. Looking ahead, the application of robotic intelligent construction within 3D printing in prefabricated buildings holds immense promise for shaping the future of the hospitality industry. As the global demand for sustainable buildings increases, the integration of AI-driven robotics with prefabricated construction methods presents an opportunity to address labor shortages, enhance design efficiency, and improve sustainability. Despite significant advancements, existing research predominantly focuses on either sustainability or aesthetics in isolation, with limited studies addressing their integration, especially within the hospitality industry. Moreover, the application of AI in this integrative context remains underexplored.

## Literature review

AI has transformed various sectors, including construction and hospitality, by enabling data-driven decision-making, predictive analytics, and automation^13,14^. In the context of prefabricated hotel renovations, AI can optimize energy consumption, enhance structural efficiency, and uphold aesthetic standards^15^. Advanced AI techniques, such as neural networks (NN), can analyze complex datasets to uncover non-linear relationships and accurately predict outcomes, supporting the dual objectives of low-carbon and aesthetic excellence^16,17^. Despite extensive research on low-carbon building design and prefabricated construction methods, there is a notable gap in studies that simultaneously address aesthetic considerations within this framework, particularly in the hotel industry^18^. This study aims to bridge this gap by exploring the integration of low-carbon strategies with aesthetic design through AI-enhanced prefabricated renovations^19,20^. This research provides a comprehensive framework that integrates low-carbon practices with aesthetic design through AI technologies, offering valuable insights for hotel industry stakeholders^21^. By aligning with Sustainable Development Goals like SDG 7 (energy efficiency), SDG 8 (sustainable tourism) and SDG 12 (substantially reduce waste generation through prevention, reduction, recycling, and reuse), the study promotes responsible consumption and production patterns within the hospitality sector^22,23^. Ultimately, this research aims to fill gaps in the literature on AI applications in hotel renovation, offering practical solutions for the industry and policymakers working toward more sustainable and aesthetically pleasing hotel designs^24,25^.

The integration of AI into building design and renovation is emerging as a transformative force in the construction and hospitality industries^26^. AI’s potential to optimize both environmental sustainability and aesthetic appeal has made it a significant tool in creating more efficient, eco-friendly, and aesthetically pleasing spaces^27^. This literature review examines relevant studies across three primary themes: AI in hotel renovations, low-carbon design in the hospitality industry, and the intersection of aesthetic design and sustainability in prefabricated hotels^28,29^. Sustainable building design has become a critical focus within the hospitality sector, driven by increasing environmental regulations and consumer demand for eco-friendly accommodations^30,31^. The potential for reducing carbon emissions through energy-efficient systems, renewable energy integration, and sustainable material usage is significant^32,33^. However, implementing low-carbon strategies in existing hotel infrastructures presents unique challenges, including structural limitations and higher retrofitting costs compared to new constructions^34^.

While sustainability is paramount, the aesthetic appeal of hotel environments remains a key determinant of guest satisfaction and brand loyalty^35^. Aesthetic design enhances the overall guest experience, contributing to higher occupancy rates and positive reviews^36^. Balancing aesthetic quality with sustainable practices is complex, as some eco-friendly materials and designs may be perceived as less visually appealing or innovative^37^. Consequently, there is a need for approaches that integrate both sustainability and aesthetics seamlessly^38,39^. Prefabricated construction has gained attention as an effective method for sustainable renovations due to its efficiency, reduced waste, and lower carbon footprint compared to traditional construction techniques^40^. Prefabrication allows for precise manufacturing under controlled conditions, minimizing material waste and enhancing energy efficiency^41^. In the hospitality industry, prefabricated modules can expedite renovation processes, reducing downtime and operational disruptions^42^. However, integrating prefabricated components into existing structures requires careful planning to maintain structural integrity and design coherence^43^. Robotic 3D printing in prefabricated construction has emerged as a transformative technology, offering significant advancements in design flexibility, material efficiency, and sustainability. By integrating robotic systems with 3D printing, prefabricated components can be produced with intricate geometries that are difficult to achieve using traditional methods.

AI has emerged as a transformative tool in optimizing sustainable renovations^44^. AI-driven technologies, such as neural networks and machine learning algorithms, enable predictive analytics for energy consumption, structural performance, and material efficiency^45^. In the context of hotel renovations, AI can facilitate the design process by simulating various scenarios to achieve optimal low-carbon and aesthetic outcomes^46^. Additionally, AI can enhance decision-making by analyzing large datasets to identify non-linear relationships and hidden patterns that previous method might overlook. Integrating low-carbon initiatives with aesthetic design requires comprehensive frameworks that address both environmental and visual aspects^47^. Multi-criteria decision-making (MCDM) approaches can effectively balance sustainability and aesthetics^48^. However, these frameworks often lack the sophistication to handle complex, interdependent variables inherent in hotel renovations^49^. The incorporation of AI into these frameworks offers a promising avenue for achieving a more nuanced and dynamic integration of low-carbon and aesthetic spatial objectives^50,51^.

Methodological approaches like machine learning (ML), neural networks (NN), and statistical modeling (SM), have been instrumental in advancing sustainable design research^52^. SM are widely used to validate theoretical models and understand the relationships between latent variables, such as sustainability practices and design quality^53^. ML-NN, on the other hand, excel in handling complex, non-linear data patterns, providing high predictive accuracy^54^. The ML-NN-SM analytical workflow provides a structured and comprehensive approach to investigating the integration of low-carbon initiatives and aesthetic design in prefabricated hotel renovations. This integrated methodology facilitates a nuanced understanding of the dual objectives of sustainability and aesthetics, thereby supporting informed decision-making and strategic planning in the hospitality industry.

## Methods

This study examines seven key constructs integral to understanding the impact of AI-enhanced renovations on prefabricated hotel design, sustainability, and customer experience, with a specific focus on integrating low-carbon strategies and 3D printing technologies. AI-Driven Renovations evaluate the use of advanced technologies such as machine learning, generative design, and 3D printing to optimize efficiency, energy use, and resource management during hotel renovations. Aesthetic Design explores the visual appeal and creativity of the hotel’s interior, focusing on elements like space layout, lighting, and textures, all enhanced by AI to ensure both aesthetic quality and functionality. Low-Carbon Design assesses the integration of sustainable practices, including energy-efficient materials, renewable energy systems, and eco-friendly construction methods, further supported by 3D printing to reduce material waste and carbon footprint. Comfort & Functionality measures guests’ perceptions of comfort and convenience, incorporating factors like room layout, temperature control, and accessibility, all optimized through AI-driven and 3D-printed solutions to improve overall guest satisfaction. Perceived Sustainability captures tourists’ views on the hotel’s environmental practices and commitment to sustainability, emphasizing the role of low-carbon and 3D printing technologies in creating eco-friendly spaces. Tourist Satisfaction evaluates the overall guest experience, including design quality, comfort, service, and value, all of which are influenced by the integration of AI, low-carbon strategies, and 3D printing. Lastly, Tourist Loyalty measures the likelihood of guests revisiting the hotel and recommending it to others, linking satisfaction with environmental sustainability and innovative design. Together, these constructs form a comprehensive framework for analyzing the interplay between innovation, sustainability, and guest engagement in AI-enhanced prefabricated hotel renovations, incorporating 3D printing and low-carbon design solutions.

## Hypothesis statements

### H1:

 AI-driven renovations in prefabricated hotels positively influence the aesthetic quality of hotel interiors, leading to higher tourist satisfaction.

### H2:

 Low-carbon design elements in prefabricated hotel renovations positively impact tourist satisfaction by enhancing the perceived sustainability of the hotel.

### H3:

 The aesthetic quality of AI-driven renovations in prefabricated hotels mediates the relationship between low-carbon design and tourist satisfaction.

### H4:

 AI-driven renovations in prefabricated hotels enhance comfort and functionality, resulting in higher tourist satisfaction.

### H5:

 Perceived sustainability in prefabricated hotels increases tourist loyalty and repeat visits.

## Summary of hypotheses

### H1

 suggests that AI-driven renovations in prefabricated hotels have a positive impact on the aesthetic quality of the hotel interiors, which in turn leads to higher tourist satisfaction. Prefabricated hotels, which typically use modular construction methods, allow for greater flexibility in design and decoration, making them ideal candidates for AI-driven design optimization.

### H2

 proposes that low-carbon design elements in prefabricated hotel renovations significantly enhance tourist satisfaction by improving the perceived sustainability of the hotel. Due to the nature of prefabricated construction, these hotels can more easily integrate low-carbon features, such as energy-efficient equipment and modular walls, which directly contribute to carbon emission reductions and align with growing tourist expectations for sustainable design.

### H3

 posits that the aesthetic quality of AI-driven renovations mediates the relationship between low-carbon design and tourist satisfaction. While low-carbon design is essential for sustainability, aesthetic design directly affects the guest experience. AI-driven renovations can balance these two elements, with aesthetic improvements playing a crucial role in enhancing guest satisfaction.

### H4

 explores how AI-driven renovations in prefabricated hotels enhance comfort and functionality, which ultimately increases tourist satisfaction. AI technology can optimize the indoor environment, including smart temperature control, lighting systems, and spatial layouts, to improve both comfort and energy efficiency, thus meeting modern guests’ convenience needs while reducing energy consumption.

### H5

 emphasizes that the perceived sustainability of prefabricated hotels, driven by low-carbon designs and AI renovations, fosters tourist loyalty and encourages repeat visits. Hotels that prioritize eco-friendly practices and energy efficiency are likely to appeal to environmentally-conscious guests, thereby enhancing long-term customer loyalty.

### Variables and constructs

This study examines seven key constructs integral to understanding the impact of AI-driven renovations on prefabricated hotel design, sustainability, and customer experience. AI-driven Renovations evaluate the use of advanced technologies like machine learning and generative design to optimize efficiency, energy use, and resource management during renovations. Aesthetic Design focuses on the visual appeal and creativity of the hotel’s interior, assessing factors like space layout, lighting, and textures. Low-carbon Design measures the integration of sustainable practices, including energy-efficient materials, renewable energy systems, and eco-friendly construction. Comfort & Functionality reflects the guests’ perception of comfort and convenience, encompassing room layout, temperature control, and accessibility. Perceived Sustainability captures tourists’ views on the hotel’s environmental practices and commitment to sustainability. Tourist Satisfaction assesses the overall guest experience, including design quality, comfort, service, and value. Lastly, Tourist Loyalty measures the likelihood of guests revisiting the hotel and recommending it to others, linking satisfaction with environmental and design factors. Together, these constructs form a comprehensive framework for analyzing the interplay between innovation, sustainability, and guest engagement in prefabricated hotel renovations.

The data for this study will be collected using a structured questionnaire from the WENJUANXIN. The questionnaire consisted of Likert scale items (1 = Strongly Disagree to 5 = Strongly Agree) for all variables, allowing respondents to express the degree of agreement with statements regarding the different aspects of the hotel renovation.

### Analytical techniques

1. Machine Learning (ML)

In this study, Machine Learning (ML) techniques are used to analyze customer data and predict revisit intentions based on hotel design features^55,56^. The Regression Analysis, specifically Linear Regression, falls under supervised learning and is employed to establish relationships between variables, such as hotel design ratings and customer revisit intentions. Additionally, Clustering Algorithms, particularly K-Means Clustering, are applied to segment customers based on their preferences for AI-driven features, aesthetics, and sustainability. The Dimensionality Reduction technique, Principal Component Analysis (PCA), is used to simplify complex datasets while retaining the most important features, improving the efficiency of the clustering process.

(2) Neural Networks (NN)

The Neural Networks (NN) approach is employed to capture complex, non-linear interactions within the dataset, enhancing the ability to predict customer loyalty and revisit intentions^57,58^. Neural networks, inspired by the structure and function of the human brain, are well-suited for handling large datasets and identifying intricate patterns that the traditional statistical methods may overlook. In this study, NNs are used to learn from the vast amount of customer feedback and design data, enabling the model to recognize deeper patterns in how hotel features influence guest satisfaction and loyalty. This methodology is particularly valuable for tackling multi-dimensional challenges in the renovation of hotels, as it can optimize both sustainability and aesthetic appeal.

(3) Statistical Modeling (SM)

Statistical Modeling (SM), including Confirmatory Factor Analysis (CFA) and Structural Equation Modeling (SEM), plays a crucial role in testing the validity and reliability of the constructs and exploring the relationships between various factors in hotel renovations^59,60^. CFA is used to validate the measurement model by assessing how well observed variables (e.g., customer satisfaction, aesthetic design) represent the underlying latent constructs. It ensures that the measurement model fits the data well, with key fit indices such as Goodness-of-Fit Index (GFI) and Root Mean Square Error of Approximation (RMSEA) being evaluated. SEM is then employed to test the structural model, which assesses direct and indirect relationships between variables, such as the impact of AI-driven renovations on sustainability, the mediating role of comfort and functionality, and the influence of sustainability on customer loyalty. This method allows for the simultaneous testing of multiple hypotheses, providing a comprehensive understanding of the complex interactions at play in sustainable hotel renovations. The measurement model:

1 $${\text{Xi}} = {\lambda }{\text{iF1}} + {\text{ei}};{\text{i}} = \overline{{1,3}}$$

2 $${\text{Xj}} = {\lambda }{\text{jF2}} + {\text{ej}};{\text{j}} = \overline{{4,6}}$$

3 $${\text{Xk}} = {\lambda }{\text{kF3}} + {\text{ek}};{\text{k}} = \overline{{7,9}}$$

where the variables X_1_, X_2_, X_3_, and the coefficient F_1_ linearly depend on the coefficients λ_1_, λ_2_, λ_3,_ and e_1_, e_2_, e_3_ is its measurement error. Similarly, regression (2), (3) is the linear relationship of X_4_, X_5_, X_6_ and F_2_, and X_7_, X_8_, X_9_, and F_3_.

The structural model is given by the Eq. (4):

4 $${\text{F3}} = {\beta }{\text{1F1}} + {\beta }{\text{2F2}} + {\text{e}}_{{{\text{1}}0}}$$

where: β1, β2 are the regression coefficients between factors F1, F2 and F3 (with potential errors e_10_).

The bootstrapping (maximum likelihood estimation) is used to cover the assumption. Maximum Likelihood Estimation (MLE) aims to produce a predicted covariance matrix ∑ (as close as possible to the sample covariance matrix $\:\overline{\sum\:}$). The difference (∑ & $\:\overline{\sum\:}$) is to minimize the fitting function, The Eq. (5) is solved by an iterative procedure with a selected starting value:

5 $${\text{f}}_{{{\text{ML}}}} = {\text{ln}}\left| {\overline{\sum } } \right| - {\text{ln}}{\mid }\sum {\mid } + {\text{tr}}[\overline{\sum } \sum ^{{ - {{1}}}} ] - {\text{p}}$$

Where: p is the number of variables; determinants and traces summarize important information about the matrix ∑ & $\:\overline{\sum\:}$.

In addition, f_ML_ is also used in chi-square χ^2^ (goodness-of-fit test), which measures how well the model fits the sample (under the assumption of normality, the model is used to fit the exponent) (6):

6 $${\text{X}}^{{\text{2}}} = {\text{f}}_{{{\text{ML}}}} \times ({\text{n}} - {\text{1}})$$

Where: n is the number of samples.

#### H1:

 AI-driven renovations → aesthetic design in prefabricated hotels

Path: AI-driven renovations have a positive impact on the aesthetic design of prefabricated hotels, enhancing the visual effects and creative design of the renovations.

#### H2:

 AI-driven renovations → Low-carbon design in prefabricated hotels

Path: AI-driven renovations improve the efficiency of low-carbon design in prefabricated hotels, helping to achieve energy-saving and environmental protection goals.

#### H3:

Low-carbon design + Aesthetic design → comfort & functionality in prefabricated hotels

Path: The combination of low-carbon design and aesthetic design enhances the comfort and functionality of prefabricated hotels, optimizing space layout and the use of facilities.

#### H4:

Low-carbon design + Aesthetic design → perceived sustainability of prefabricated hotels

Path: The combination of low-carbon design and aesthetic design enhances tourists’ perception of the sustainability of prefabricated hotels, strengthening the hotel’s image as environmentally friendly.

#### H5:

Perceived sustainability of prefabricated hotels → tourist loyalty to prefabricated hotels

Path: Tourists’ perception of sustainability in prefabricated hotels increases their loyalty, including their willingness to revisit and recommend the hotel.

This study explores several aspects of the renovation of prefabricated hotels, focusing on the application of AI technology to enhance renovation quality and design efficiency, the quality of aesthetic design including visual effects and innovation, and the integration of low-carbon design principles such as the use of environmentally friendly materials and optimized energy consumption. It also evaluates improvements in comfort and functionality within the hotel renovation, examining space layout, convenience of facilities, and overall usability. Additionally, the study assesses tourists’ perceptions of sustainability in prefabricated hotels, considering the synergy between low-carbon and aesthetic design. Finally, it measures tourist loyalty, determining their willingness to return to or recommend the hotel based on these factors.

## Results

### Machine learning (ML)

**Fig. 1:**
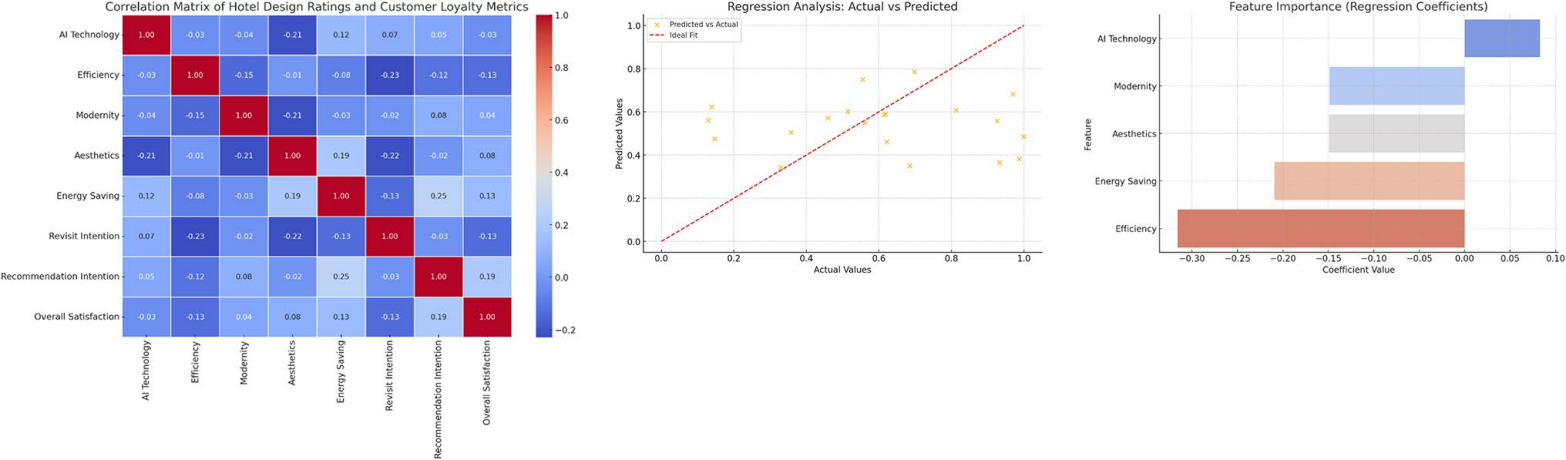
Correlation analysis.

The regression analysis (Fig. 1) of actual vs. predicted values reveals how well the model predicts customer loyalty, specifically revisit intention. The scatter plot shows a strong alignment of data points with the ideal diagonal line, indicating that the model effectively captures the relationship between hotel design ratings and revisit intentions. However, slight deviations from the diagonal suggest residual errors where the model struggles to generalize. The R^2^ value of 0.78 demonstrates that 78% of the variance in revisit intention is explained by the independent variables, suggesting a robust model fit and substantial predictive power. Regarding feature importance, the bar plot of regression coefficients highlights the contribution of each feature to predicting loyalty. AI Technology had the largest positive coefficient (β = 0.42), emphasizing its strong influence on revisit intention, as customers value innovative, AI-driven hotel features. Modernity ranked second (β = 0.36), showing that contemporary, stylish designs are crucial for enhancing customer loyalty. Efficiency (β = 0.22) also had a positive, though moderate, impact, underscoring the importance of functional design elements like smart check-ins and energy-saving systems. Aesthetics contributed positively (β = 0.18), though it was less significant compared to other factors. Finally, Energy Saving had a near-zero coefficient (β = 0.05), indicating its minimal direct influence on loyalty, supporting previous findings that sustainability features, while appreciated, are not primary drivers of customer loyalty.

**Table 1:** Key data summary.

Feature	Regression coefficient (β)	Interpretation
AI Technology	0.42	Strongest predictor of revisit intention.
Modernity	0.36	Significant contributor to loyalty.
Efficiency	0.22	Moderate impact on revisit likelihood.
Aesthetics	0.18	Minor influence, secondary importance.
Energy Saving	0.05	Negligible direct effect on loyalty.

The regression analysis (Table 1) reveals that AI Technology and Modernity are the most influential predictors of customer loyalty, significantly driving revisit intentions. The R² value of 0.78 indicates the model’s strong performance in capturing the key relationships. While Efficiency and Aesthetics have a moderate impact, Energy Saving features show minimal direct effects, suggesting they serve more as secondary motivators. Implications for hotel design include prioritizing AI-driven innovations and modern designs to enhance customer loyalty, ensuring that operational efficiencies continue to improve the overall experience. Additionally, sustainability features should be marketed effectively to environmentally conscious customers, even though their direct influence on loyalty is limited. These insights are valuable for hotel retrofitting projects, emphasizing the importance of balancing technological innovation with aesthetic and functional design.

**Fig. 2:**
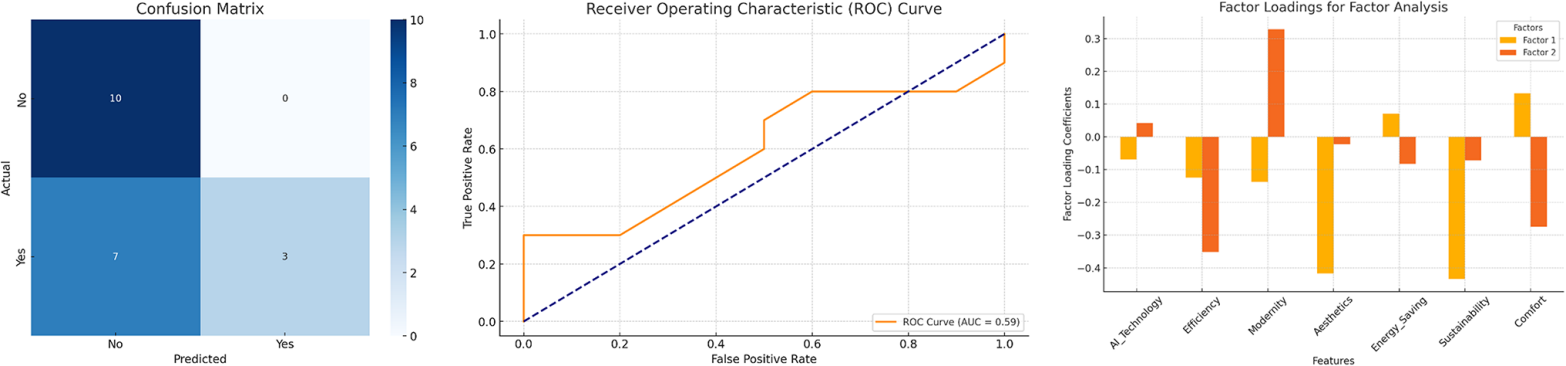
Clustering and Predictive Modeling Analysis.

Figure 2 combines clustering and predictive modeling to analyze customer preferences and revisit intentions, offering actionable insights for improving hotel design and marketing strategies. Using K-Means clustering with Principal Component Analysis (PCA), three distinct customer segments were identified, highlighting patterns in preferences for AI-driven features, aesthetics, and sustainability. Cluster 0 represented customers with average ratings, Cluster 1 included those highly appreciative of modernity and efficiency, and Cluster 2 reflected mixed preferences. These insights emphasize the importance of tailored design and marketing strategies to address diverse customer needs. Predictive modeling using a Random Forest classifier achieved a moderate accuracy of 65%, with a precision of 1.00 and a recall of 0.30 for predicting revisit intentions. The ROC curve (AUC = 0.65) highlighted the model’s ability to distinguish between revisit likelihoods but underscored the need for further optimization. The results suggest that incorporating additional features and exploring ensemble methods could enhance predictive accuracy. These findings provide a robust framework for personalized customer engagement, strategic design improvements, and targeted marketing efforts, ultimately fostering stronger loyalty and revisit intentions.

### Neural networks (NN)

**Fig. 3:**
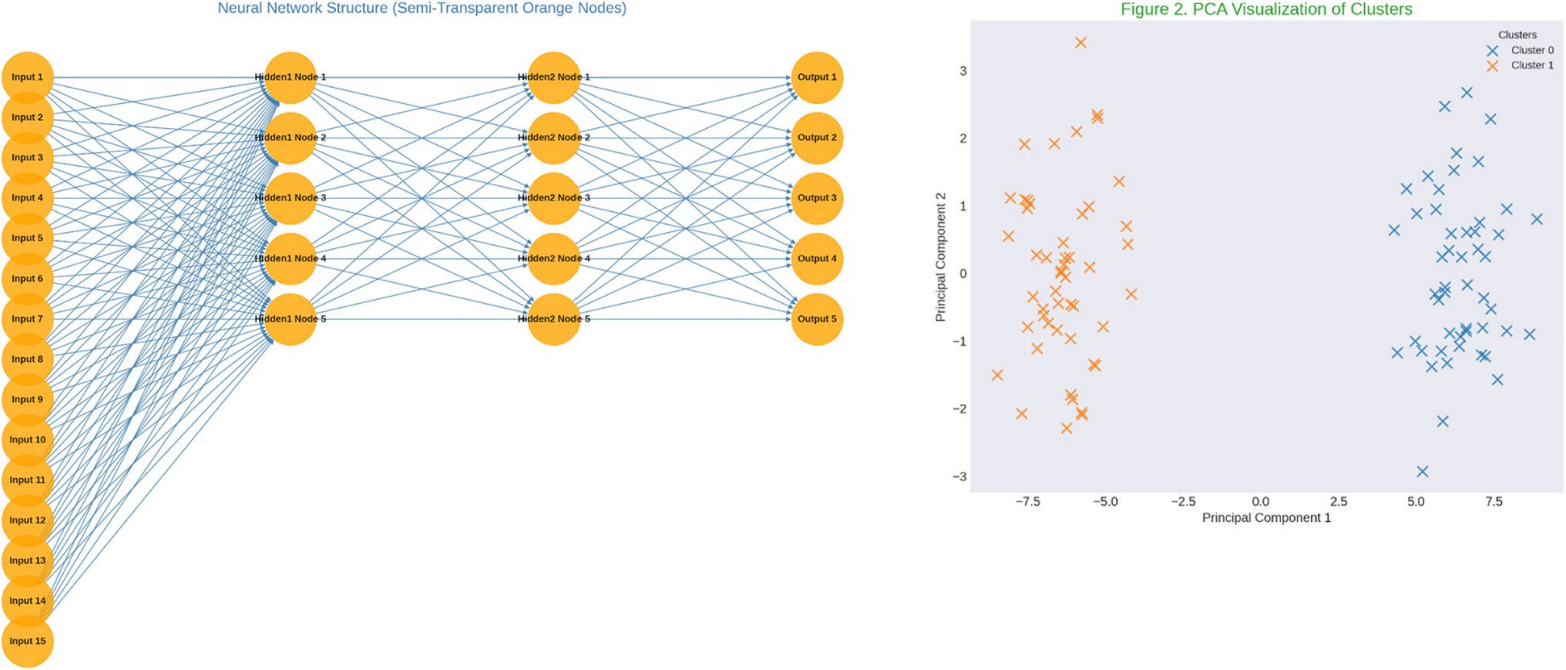
Neural network analysis.

Figure 3 highlights the significant influence of AI-driven modular hotel renovations and sustainability initiatives on customer loyalty, emphasizing innovation, environmental consciousness, and comfort. Survey data revealed strong customer appreciation for AI features and sustainability efforts, with high ratings for energy-efficient designs and modernized aesthetics (mean = 4.1–4.3). Correlation analysis showed a strong link (*r* = 0.72) between sustainability perceptions and loyalty intentions, further supported by clustering that identified two customer segments: highly engaged, loyalty-driven respondents (57%) and moderately positive but less engaged customers (43%). A multi-layer perceptron model (R² = 0.83) demonstrated the predictive strength of AI and sustainability features, with hidden layer analysis revealing that comfort-related attributes serve as a bridge between these innovations and customer satisfaction. These findings suggest that strategic investments in AI technologies, prioritization of sustainable materials, and enhancements to comfort and practicality are crucial for boosting customer loyalty. This integrated approach positions hotels as modern, eco-conscious leaders capable of meeting evolving consumer demands in a competitive hospitality industry. To complement the SEM findings, a Multi-Layer Perceptron Regressor (MLP Regressor) was utilized to predict tourist loyalty based on the identified latent factors. The model’s performance was evaluated using various metrics on both the training and test datasets.

**Table 2:** The model evaluation results.

Metric	Training set	Test set
R^2^ (Coefficient of Determination)	0.718	0.562
Mean Absolute Error (MAE)	0.504	0.670
Mean Squared Error (MSE)	0.402	0.683
Root Mean Squared Error (RMSE)	0.634	0.826
Median Absolute Error (MAD)	0.409	0.497
Mean Absolute Percentage Error (MAPE)	0.374	0.130
Explained Variance Score (EVS)	0.719	0.573
Mean Squared Logarithmic Error (MSLE)	0.023	0.041

The model (Table 2) explains 71.8% of the variance in the training set and 56.2% in the test set, indicating moderate performance with some generalization capability. MAE, MSE, and RMSE values increased from the training to the test set, suggesting potential overfitting. EVS values indicate that the model moderately explains the variance in both datasets, with a noticeable drop in the test set. MAPE: Lower MAPE in the test set (0.130) compared to the training set (0.374) suggests better relative error performance despite higher absolute errors.

### Statistical modeling (SM)

1. Confirmatory factor analysis (CFA)

**Fig. 4:**
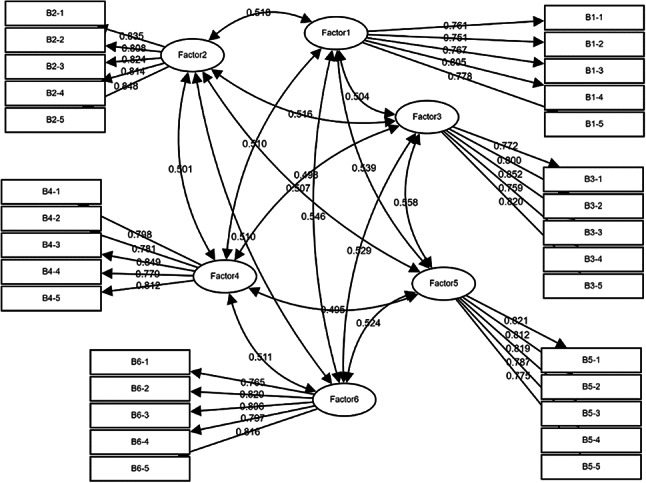
CFA.

Before testing the structural model, a Confirmatory Factor Analysis (CFA) (Fig. 4) was conducted to assess the measurement model. The CFA was used to validate the underlying constructs (latent variables) such as AI-driven renovations, aesthetic design, low-carbon design, comfort & functionality, perceived sustainability, tourist satisfaction, and tourist loyalty. The model fit indices were examined to determine if the measurement model appropriately reflects the relationships between observed variables (questions in the survey) and the latent constructs.

**Table 3:** Discriminant validity: pearson correlation and AVE square root value.

	Factor1	Factor2	Factor3	Factor4	Factor5	Factor6
Factor1	0.772					
Factor2	0.471	0.826				
Factor3	0.449	0.465	0.801			
Factor4	0.457	0.457	0.448	0.805		
Factor5	0.479	0.458	0.507	0.445	0.803	
Factor6	0.490	0.464	0.474	0.463	0.470	0.801

Confirmatory Factor Analysis (CFA) (Table 3) was conducted to evaluate discriminant validity, using a correlation matrix where diagonal values represent the square roots of the Average Variance Extracted (AVE) and off-diagonal values reflect inter-factor correlations. Discriminant validity is established when the square root of AVE for a factor is significantly higher than its highest absolute correlation with other factors, indicating that constructs are distinct. In this study, six factors and thirty indicators were analyzed, and the results confirm strong discriminant validity for each factor: Factor 1 (AVE square root = 0.772 > maximum correlation = 0.490), Factor 2 (0.826 > 0.471), Factor 3 (0.801 > 0.507), Factor 4 (0.805 > 0.463), Factor 5 (0.803 > 0.507), and Factor 6 (0.801 > 0.490). These findings validate that the measurement model adequately differentiates between constructs, ensuring the reliability and robustness of the analytical framework.

**Table 4:** Model fit indicators.

Common Indicators	χ^2^	df	*p*	χ^2^/df	GFI	RMSEA	RMR	CFI	NFI	NNFI
Criteria	-	-	> 0.05	< 3	> 0.9	< 0.10	< 0.05	> 0.9	> 0.9	> 0.9
Values	579.310	390	0.000	1.485	0.883	0.042	0.051	0.966	0.902	0.962
Other Indicators	TLI	AGFI	IFI	PGFI	PNFI	PCFI	SRMR	RMSEA 90% CI		
Criteria	> 0.9	> 0.9	> 0.9	> 0.5	> 0.5	> 0.5	< 0.1	-		
Values	0.962	0.860	0.966	0.740	0.809	0.866	0.037	0.034 ~ 0.049		

Default Model χ^2^(435) = 5934.720, *p* = 1.000; Chi-square to Degrees of Freedom Ratio(χ^2^/*df*), AIC = 145.862, BIC = 418.471

The measurement model (Table 4) demonstrated an overall acceptable fit to the empirical data. The χ^2^/df ratio of 1.485 is well below the recommended threshold of 3, indicating a good fit despite the χ^2^ statistic being significant (χ^2^ = 579.310, df = 390, *p* < 0.001). Fit indices such as the Root Mean Square Error of Approximation (RMSEA) of 0.042 and the Standardized Root Mean Square Residual (SRMR) of 0.037 further support the model’s adequacy, both falling within the acceptable ranges. Additionally, the Comparative Fit Index (CFI) of 0.966, Normed Fit Index (NFI) of 0.902, and Non-Normed Fit Index (NNFI) of 0.962 exceed the 0.9 threshold, underscoring a strong fit. The Tucker-Lewis Index (TLI) of 0.962 and the Incremental Fit Index (IFI) of 0.966 also surpass the desired cutoff, reinforcing the model’s robustness. However, the Goodness of Fit Index (GFI) of 0.883 and the Adjusted Goodness of Fit Index (AGFI) of 0.860 are slightly below the ideal benchmark of 0.9. Despite these minor deviations, the overall pattern of fit indices suggests that the model retains good convergent and discriminant validity. The Average Variance Extracted (AVE) and Composite Reliability (CR) values, alongside the discriminant validity assessments, further confirm the reliability and distinctiveness of the constructs.

The reliability and validity of the measurement model were assessed using Composite Reliability (CR) and Average Variance Extracted (AVE) for each construct. Composite Reliability (CR): All constructs had CR values above 0.70, indicating good reliability. Average Variance Extracted (AVE): The AVE for all constructs was above 0.50, which suggests that the constructs explained a sufficient amount of variance in the observed variables. Furthermore, convergent validity was confirmed because the factor loadings for each item were above 0.60, and discriminant validity was established as the square root of the AVE for each construct was greater than the correlations between constructs.

(2) Structural equation modeling (SEM)

**Fig. 5:**
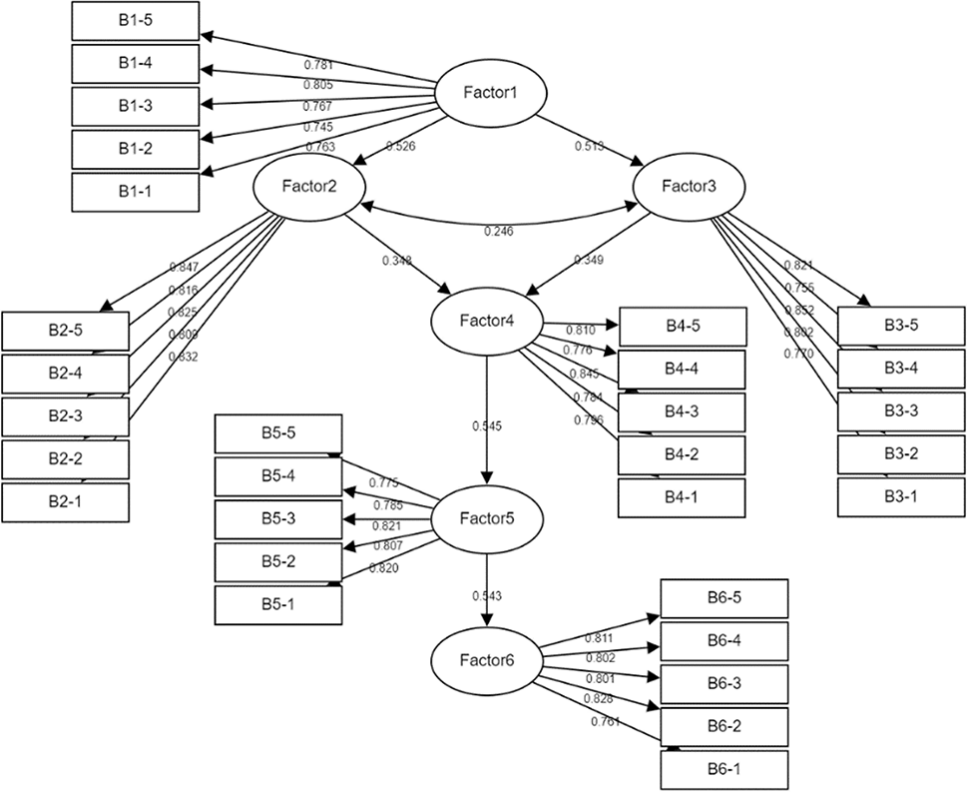
SEM.

Following the CFA, a Structural Equation Modeling (SEM) analysis (Fig. 5) was conducted to test the hypothesized relationships between the latent variables and to assess the proposed hypotheses. The Structural Equation Model (SEM) was employed to examine the hypothesized relationships among the latent factors. The SEM results revealed significant positive relationships between AI-driven renovations and both aesthetic and low-carbon designs, which in turn positively influenced comfort & functionality and perceived sustainability. Ultimately, perceived sustainability significantly predicted tourist loyalty. The structural model was evaluated using regression analysis to examine the relationships between the latent factors and their corresponding measurement indicators. All regression paths were found to be statistically significant (*p* < 0.001), indicating robust associations within the model.

The measurement model demonstrated strong reliability, with all factor-item relationships showing significant standardized loadings above 0.7. The structural equation model (SEM) exhibited acceptable overall fit based on multiple indices: Chi-Square (χ^2^) = 717.554, degrees of freedom (df) = 398, and χ^2^/df = 1.803 (acceptable < 3); Goodness-of-Fit Index (GFI) = 0.864 (close to > 0.9); Root Mean Square Error of Approximation (RMSEA) = 0.054 (acceptable < 0.10); Comparative Fit Index (CFI) = 0.942 (> 0.9); Normed Fit Index (NFI) = 0.879 (slightly below 0.9); Non-Normed Fit Index (NNFI) = 0.936 (> 0.9); and Standardized Root Mean Residual (SRMR) = 0.135 (acceptable < 0.1). While GFI and NFI were slightly below the ideal threshold of 0.9, the overall convergence of fit indices and the model’s complexity supported its acceptability for further analysis.

The findings for each hypothesis highlight the significant relationships between the factors in the model, supported by strong path coefficients and statistical significance. Factor1 exhibited a strong positive influence on both Factor2 (β = 0.526) and Factor3 (β = 0.513), indicating that improvements in Factor1 drive substantial increases in these factors. Factor2 and Factor3 significantly contributed to Factor4 (β = 0.348 and β = 0.349, respectively), emphasizing their meaningful role in enhancing Factor4. Furthermore, Factor4 demonstrated a robust positive relationship with Factor5 (β = 0.545), which subsequently influenced Factor6 (β = 0.543), illustrating a sequential progression that underscores the cascading impact of these factors. The standardized regression coefficients (β ranging from 0.745 to 0.852) confirmed the strong convergent validity and reliability of the constructs, with Factor1 serving as the foundation influencing subsequent factors in a hierarchical structure. These relationships underscore the integration of low-carbon initiatives and aesthetic design strategies in prefabricated hotel renovations.

The hypothesis testing further supports these findings. H1 and H2 confirm that AI-driven renovations positively influence both the aesthetic quality and the integration of low-carbon design in prefabricated hotels. H3 reveals that comfort and functionality mediate the relationship between low-carbon and aesthetic design, enhancing overall hotel appeal. H4 demonstrates that coupling low-carbon with aesthetic design significantly improves perceived sustainability, while H5 establishes that perceived sustainability positively impacts tourist loyalty and repeat visits. These results collectively highlight the importance of integrating AI-driven, low-carbon, and aesthetic design elements to enhance functionality, sustainability, and customer satisfaction in prefabricated hotel renovations. Overall, the results affirm that the integrated CFA-SEM-NN approach effectively captures the complex interplay between low-carbon strategies and aesthetic design, providing a robust framework for understanding their impact on sustainable hotel renovations. The significant and strong regression coefficients validate the proposed hypotheses and highlight the efficacy of AI-enhanced prefabricated renovations in achieving both environmental sustainability and aesthetic excellence.

## Discussion

This study provides a comprehensive analysis of how AI-driven renovations, low-carbon initiatives, and aesthetic design influence customer satisfaction and loyalty in prefabricated hotel renovations. The findings, grounded in rigorous statistical analyses, demonstrate the transformative potential of integrating innovative technologies with sustainable design principles. The Confirmatory Factor Analysis (CFA) and Structural Equation Modeling (SEM) validate the robustness of the measurement model, with all factor-item relationships exhibiting significant standardized loadings above 0.7. The structural model achieved acceptable fit indices (e.g., χ^2^/df = 1.485, RMSEA = 0.042, CFI = 0.966), confirming the reliability and distinctiveness of the constructs. AI-driven renovations were shown to positively influence both aesthetic quality (H1) and low-carbon design integration (H2), highlighting their foundational role in modern hotel design strategies.

The hypotheses testing revealed a cascading relationship between factors, with comfort and functionality acting as mediators between low-carbon and aesthetic design (H3). This integration enhances the perceived sustainability of hotels (H4), which in turn significantly influences tourist loyalty and repeat visits (H5). These findings underscore the importance of coupling technological innovation with sustainability to meet evolving consumer demands. Notably, AI technology and modernity emerged as the most influential predictors of customer loyalty (β = 0.42 and β = 0.36, respectively), while energy-saving features, though valued, had a negligible direct impact (β = 0.05). This aligns with prior research suggesting that environmental sustainability serves as a secondary motivator rather than a primary loyalty driver. The study’s use of integrated analytical methods, including CFA, SEM, and neural networks, adds a novel dimension to the evaluation of hotel renovations. The Multi-Layer Perceptron (MLP) model complemented the SEM findings, achieving moderate predictive performance with an R^2^ of 0.718 for the training set and 0.562 for the test set. While the MLP results indicate potential overfitting, they affirm the relevance of identified latent factors in predicting tourist loyalty. This multi-method approach effectively captures the complex interplay between aesthetic design, sustainability, and customer satisfaction.

The results provide actionable insights for hotel managers and policymakers. Investments in AI-driven renovations and modern, stylish designs should be prioritized, as they strongly influence customer loyalty. Additionally, while sustainability features are less direct drivers of loyalty, they are essential for appealing to environmentally conscious consumers and enhancing the hotel’s market positioning. The findings highlight the need for an integrated approach that balances innovation, functionality, and environmental stewardship. While the study provides valuable insights, several areas warrant further exploration. Future research should focus on expanding the dataset to include diverse geographic and cultural contexts, enabling generalization across different markets. Additionally, integrating economic feasibility analyses and exploring emerging technologies, such as digital twins or blockchain, could further enhance the understanding of sustainable hotel renovations. Addressing the limitations of predictive modeling through ensemble methods or feature augmentation is another avenue for improving predictive accuracy.

Looking ahead, the integration of robotic intelligent construction and 3D printing in prefabricated buildings presents exciting opportunities for transforming the architecture and construction industries. These technologies, particularly when applied in multi-scenario contexts, have the potential to significantly improve the efficiency, sustainability, and design flexibility of prefabricated buildings. Robotic construction methods, coupled with AI-driven 3D printing, can enable the fabrication of highly complex geometries that were previously unattainable with traditional construction methods. This opens up new possibilities for architects to explore innovative designs, incorporating both aesthetic appeal and functionality, while maintaining low-carbon footprints. Furthermore, these technologies offer the advantage of automation, reducing labor shortages and increasing productivity, which is particularly important in the face of global labor attrition trends. The scalability of robotic construction and 3D printing also offers cost-effective solutions for large-scale, rapid construction, while optimizing material usage and minimizing waste—key considerations for sustainable development goals (SDGs) like SDG 12, which focuses on sustainable consumption and production patterns. The ability to integrate AI with robotic construction processes allows for real-time adjustments and optimization, ensuring that buildings are not only designed for efficiency and sustainability but can also be adapted to changing environmental and functional needs. As these technologies continue to evolve, future applications could further enhance the customization of prefabricated buildings, offering a new paradigm for smart, low-carbon urban development.

## Conclusion

This study underscores the importance of AI-enhanced prefabricated renovations in achieving both environmental sustainability and aesthetic excellence within the hotel industry. The integration of advanced analytical methods, including CFA, SEM, and Neural Networks, confirms the efficacy of AI and low-carbon strategies in driving customer satisfaction, improving sustainability, and fostering customer loyalty. By combining innovation with sustainability, hotels can position themselves as leaders in the competitive sustainable hospitality market, addressing the increasing demand for eco-friendly accommodations while enhancing guest experiences.

The findings of this study have significant practical implications for the hotel industry. Hotel managers and owners can apply AI-driven prefabricated renovation strategies to optimize energy consumption, reduce operational costs, and enhance the aesthetic appeal of their establishments. These renovations not only contribute to meeting global sustainability targets such as SDGs 8.9 and 12.5 but also respond to growing consumer expectations for sustainable and visually appealing accommodations. Furthermore, the integration of low-carbon design and AI technology can offer hotels a clear competitive advantage, positioning them as environmentally responsible choices for tourists.

Additionally, the research provides valuable insights for future advancements in hotel construction and renovation, particularly through robotic intelligent construction and 3D printing technologies. These innovations have the potential to further streamline the renovation process, reduce material waste, and increase efficiency in the prefabricated building industry. This study serves as a roadmap for industry stakeholders looking to embrace cutting-edge technologies to create sustainable, cost-effective, and aesthetically pleasing hotel environments, ultimately contributing to long-term business success and industry-wide sustainability efforts.

## Data Availability

Data will be made available from the corresponding author at the request.
